# Complete mitochondrial DNA sequence of Golden trevally *Gnathanodon speciosus* (Forsskål, 1775) and the phylogenetic analysis of Carangidae

**DOI:** 10.1080/23802359.2022.2095231

**Published:** 2022-07-25

**Authors:** Fangyan Jiang, Ning Yang, Hai Huang

**Affiliations:** a Key Laboratory of Utilization and Conservation for Tropical Marine Bioresources of Ministry of Education, Hainan Tropical Ocean University, Sanya, China; b Key Laboratory of Tropical Marine Fishery Resources Protection and Utilization of Hainan Province, Hainan Tropical Ocean University, Sanya, China

**Keywords:** Mitochondrial genome, phylogenetic analysis, *Gnathanodon speciosus*

## Abstract

In this study, the complete mitochondrial genome of *Gnathanodon speciosus* was determined. The entire mitochondrial DNA sequence is 16,555 bp in length and consists of 13 protein-coding genes (PCGs), 22 transfer RNA (tRNA) genes, two ribosomal RNA (rRNA) genes, and a control region. Phylogenetic analysis was performed using 13 PCGs showing that *G. speciosus* belongs to the Carangidae family, and is most closely related to the species in genera *Caranx* and *Megalaspis*.

The Golden trevally, *Gnathanodon speciosus* (Forsskål, 1775) occurs throughout the tropical Indo-Pacific eastward to the Americas and is found inshore in association with rocky areas, deep lagoons, and seaward reefs (Grandcourt et al. 2004). They are bottom feeders, consuming molluscs and crustaceans, but also feed on small schooling fishes near surface waters (Elise 2011). Golden trevally is an ideal candidate species for aquaculture diversification, particularly in the Asia-Pacific region (Ranjan 2017). Additionally, it is exploited in the food industry, while juveniles are used in recreational fisheries.

In this study, the Golden trevally sample was collected from Sanya, Hainan, China (18°16′45″N, 109°30′00″E). The total length is about 26.5 cm. The fish is yellowish-green dorsally and pale yellow ventrally. There is one oblique band above the eye, and alternate broad and narrow bands on the body. Pectoral fins were stored in 75% ethanol at 4 °C. Genomic DNA was extracted using the Animal Genome Extraction Kit (Invitrogen, Waltham, MA) following the manufacturers’ instructions, and stored at −20 °C for the subsequent analysis. The specimen and DNA are stored in the museum of Hainan Tropical Ocean University (Dr. Huimin Feng, womenfeng@163.com) under voucher no. 2020G06. Our sampling procedure was approved by the Institutional Animal Care and Use Committee of Hainan Tropical Ocean University (approval ID 2020021). The mitochondrial genome was sequenced using Illumina NovaSeq 6000 sequencing (150 bp × 2, Shanghai BIOZERON Co., Ltd., Shanghai, China) (Jiang et al. 2022). The phylogenetic analysis was performed using MEGA 6. A maximum-likelihood (ML) tree was constructed under the Kimura 2-parameter model with 1000 bootstrap replicates (Tamura et al. 2013).

The complete mitogenome sequence of *G. speciosus* is 16,555 bp in length (GenBank accession no. MT922005). It exhibits a slight G + C bias of 44.23%. It has a typical mitochondrial genome structure, consisting of 13 protein-coding genes (PCGs), 22 transfer RNA genes (tRNAs), two ribosomal RNA genes (rRNAs), and a control region (CR). Except for eight tRNAs and *ND6* genes, all other mitochondrial genes were encoded on the heavy strand. The length of all tRNAs ranged from 66 to 75 bp, and their anti-codons were consistent with other fish of Carangidae. Thirteen PCGs were initiated with three types of the start codons ATC (*COX1*), ATA (*ATP6*), and the remaining genes with the ATG codon. Four types of stop codons are TAG (*ND2*, *ND3*), AGA (*COX2*), GTT (*ND6*), and the remaining genes with the TAA codon.

The phylogenetic tree for *G. speciosus* was constructed with 13 PCGs from 15 other species of Carangidae by using the ML methods. As shown in Figure 1, G. speciosus was grouped with the 12 other Caranginae species, and is most closely related to the species in genera *Caranx* and *Megalaspis*. The phylogenetic analysis supported the monophyly of the subfamily Caranginae, consistent with a previous analysis (Jeon et al. 2021). The mitogenome sequence of *G. speciosus* is helpful in the research of molecular systematics and evolutionary relationships within the family Carangidae.

**Figure 1.**
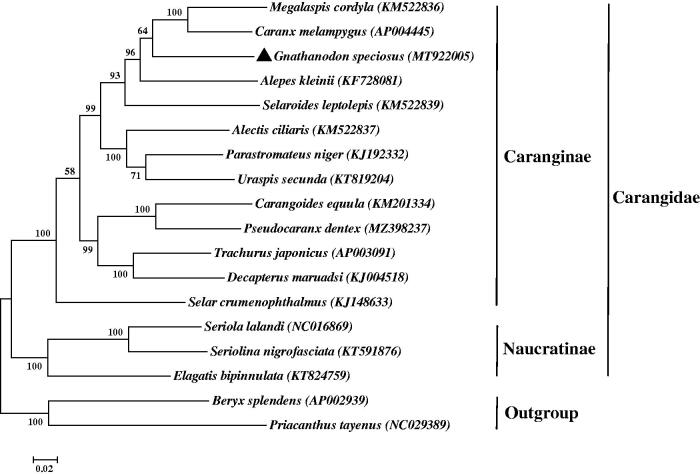
Phylogenetic relationship of the Carangidae based on 13 PCGs with 1000 bootstrap replicates. The complete mitochondrial genome sequences were downloaded from GenBank. Accession numbers are indicated in parentheses after the scientific names of each species. The number at each node is the bootstrap value. The genome sequence in this study is labeled with a black triangle.

## Data Availability

The genome sequence data that support the findings of this study are openly available in GenBank of NCBI at https://www.ncbi.nlm.nih.gov/ under the accession no. MT922005. The associated BioProject, SRA, and Bio-Sample numbers are PRJNA735568, SRR14744695, and SAMN19589873, respectively.
